# Optimizing the Magnetization-Prepared Rapid Gradient-Echo (MP-RAGE) Sequence

**DOI:** 10.1371/journal.pone.0096899

**Published:** 2014-05-30

**Authors:** Jinghua Wang, Lili He, Hairong Zheng, Zhong-Lin Lu

**Affiliations:** 1 Center for Cognitive and Behavioral Brain Imaging, The Ohio State University, Columbus, Ohio, United States of America; 2 Center for Perinatal Research, Nationwide Children’s Hospital, Columbus, Ohio, United States of America; 3 Paul C. Lauterbur Research Center for Biomedical Imaging, Shenzhen Institutes of Advanced Technology, Chinese Academy of Sciences, Shenzhen, China; Vanderbilt University, United States of America

## Abstract

The three-dimension (3D) magnetization-prepared rapid gradient-echo (MP-RAGE) sequence is one of the most popular sequences for structural brain imaging in clinical and research settings. The sequence captures high tissue contrast and provides high spatial resolution with whole brain coverage in a short scan time. In this paper, we first computed the optimal k-space sampling by optimizing the contrast of simulated images acquired with the MP-RAGE sequence at 3.0 Tesla using computer simulations. Because the software of our scanner has only limited settings for k-space sampling, we then determined the optimal k-space sampling for settings that can be realized on our scanner. Subsequently we optimized several major imaging parameters to maximize normal brain tissue contrasts under the optimal k-space sampling. The optimal parameters are flip angle of 12°, effective inversion time within 900 to 1100 ms, and delay time of 0 ms. In vivo experiments showed that the quality of images acquired with our optimal protocol was significantly higher than that of images obtained using recommended protocols in prior publications. The optimization of k-spacing sampling and imaging parameters significantly improved the quality and detection sensitivity of brain images acquired with MP-RAGE.

## Introduction

The three-dimension magnetization-prepared rapid gradient echo (MP-RAGE) sequence is one of the most popular sequences for high-resolution whole brain T_1_-weighted imaging. First proposed by Muger and Brookeman [1], the sequence combines the power of magnetization-prepared imaging and rapid 3-D gradient echo acquisition techniques to provide excellent tissue contrasts, high spatial resolution, and full brain coverage in a short scan time. Images acquired with the sequence have been widely used for classifying brain tissues in voxel-based morphometry [2], detecting pathological changes of the brain [3], [4], estimating regional brain volume abnormalities associated with brain functions [5], assessing brain development [6], and evaluating treatment or therapeutic responses [7]. In this study, we used computer simulations to optimize the MP-RAGE sequence. We addressed two issues: optimal k-space sampling and optimal imaging parameters under optimal k-space sampling. In vivo experiments showed that the quality of images acquired with our optimal protocol was significantly higher than that of images obtained using recommended protocols in prior publications. The work is important not only for image quality improvement and artifact reduction, but also for reducing the variability of images acquired across different sites and/or over time. The reduced variability is crucial for increasing the statistical power and reducing the number of required subjects in basic and clinical research.

The key objective metrics for evaluating image quality include the signal-to-noise ratio (SNR) of a desired tissue, and the contrast-to-noise ratio (CNR) between different tissues. Previous studies have shown that k-space sampling strongly affects image quality including SNR, CNR and artifacts [8]. Of particular importance is k-space center acquisition. K-space center acquisition determines the contrast of the low spatial frequency components of the k-space and is a major factor in determining tissue contrast in the image domain. Because the MR signal decays over time, k-space lines acquired at different time have different signal magnitudes. Optimizing K-space center acquisition would greatly increase image contrast. This idea is well known and has been incorporated into EPI and FSE acquisition, where the effective TE corresponding to k-space center is often optimized to achieve maximal T_2_*- or T_2_-weighted contrast. The issue has however not been considered in MP-RAGE. In fact, k-space lines acquired with different readout RF pulses in the MP-RAGE sequence have different signal amplitudes and correspond to different T_1_-weighted contrasts. Optimizing effective inversion recovery time (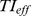) that corresponds to the k-space center would lead to increased T_1_-weighted contrast in MR images. In practice, the optimal 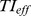 or optimal k-space center sampling varies with brain tissue properties, imaging parameters, field strength, and the number of readout RF pulses. Conventional k-space trajectories, such as sequential, centric, and reverse centric orders, constrain k-space center sampling to the beginning, center, or end of the readout RF pulses, and may result in non-optimal k-space center sampling and therefore non-optimal T_1_-weighted image contrast. In this paper, we first optimized k-space sampling by computer simulation to obtain the theoretically optimal k-space sampling. Because the k-space trajectory is rectangular and k-space sampling is set at some specific values on our scanner, we also optimized k-space sampling for the settings that can be realized on our scanner. We showed that the best choice from the limited set of options offered by the Siemens MPRAGE sequence still led to significant improvements.

We used computer simulations to optimize imaging parameters to maximize normal brain tissue contrast. Several studies have attempted to optimize the parameters of the MP-RAGE sequence for the human brain. Epstein et al. used a simulated annealing algorithm to optimize the MP-RAGE sequence [9]. Some researchers simulated SNR and CNR using Bloch’s equation to optimize imaging parameters with different conventional k-space trajectories, such as a centric phase encoding order [10], [11], 3D stack-of-rings k-space trajectory [12], and an elliptical centric view phase encoding order [13]. These optimizations focused on the effects of delay time (TD) [6], [10]–[15] and 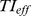 [6], [14]–[15] for images acquired with conventional k-space trajectories. In this paper, we optimize MP-RAGE imaging parameters by computer simulations and in vivo experiments under optimal k-space sampling.

We computed signal intensity and contrast of brain images by numerical simulations of Bloch’s equation with properties (T_1_, T_2_, and proton density) of the tissues of the normal human brain at 3.0 Tesla. Optimal k-space sampling was determined by the maximal simulated contrast. Optimal imaging parameters, such as flip angle, echo spacing time, delay time, and effective inversion recovery time, were obtained based on simulations under optimal k-space sampling. In vivo experiments were used to validate our simulation results. The quality of brain images acquired with optimal k-space sampling and imaging parameters was significantly improved, and outperformed that of images acquired with published imaging parameters under conventional k-space sampling.

## Materials and Methods

### The MP-RAGE Sequence

The MP-RAGE sequence is composed of 3D-inversion recovery **α** and N equally-spaced readout RF pulses of flip angle **θ** and echo spacing τ. Repetition time TR is defined as the time interval between two successive inversion recovery pulses:
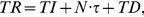
(1)where *τ* is echo spacing time, N is the total number of readout RF pulses, TI is the time interval between the inversion recovery pulse and the first RF readout pulse, and TD is delay time. In order to simplify the formula for signal intensity, we define: 
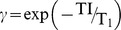
, 
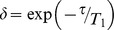
, 
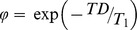
, and 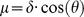. For successive excitations in the MP-RAGE sequence, signal intensity from the i^th^ read-out pulse is given by [17], [18]:
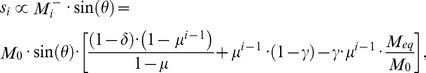
(2)where the steady state magnetization 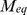 after several TRs is:



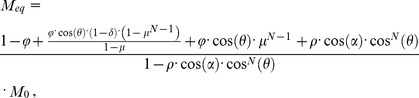
(3)The white matter (WM) and gray matter (GM) contrast from the i^th^ read-out RF pulse is given by:
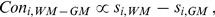
(4)Where 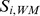 and 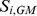 are the signal intensities of WM and GM, which can be calculated using Eq. 2 with the longitudinal relaxation times and protein densities of WM and GM, respectively. GM-WM contrast is a function of N, TI, τ, θ and the temporal position of the read-out RF pulse.

### K-Space Optimization

Although it is a property in the image domain that is determined by all Fourier components in the entire k-space, contrast between WM and GM is mostly determined by k-space center which is associated with the low spatial frequency components in k-space. According to Eqs. 2–4, GM and WM contrast from the i^th^ read-out RF pulse is a function of the temporal position of the RF pulse and the total number of read-out RF pulses N. The major objective of k-space optimization is to optimize the linear k-space trajectory such that k-space center has the maximal 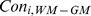.

For a sequence in a commercial scanner, the k-space strategy, including k-space trajectory and sampling order, is usually fixed to a few choices. We first computed the temporal position of the RF pulse that leads to the maximum GM-WM contrast of images acquired with the MP-RAGE sequence at 3.0 Tesla using computer simulations without considering the pre-determined scanner settings. We then determined the optimal k-space sampling for settings that can be realized on our scanner. In our scanner, we can shift k-space center by slice partial Fourier, which can be set as off, 7/8, and 6/8. When the nominal N is 176, slice partial Fourier factors of off, 7/8, and 6/8 correspond to real Ns of 176, 156, and 132, respectively. In that case, k-space center acquisition on our scanner is set to be the 88^th^, 66^th^ and 44^th^ read-out RF pulse, with corresponding middle echo at 88^th^, 78^th^ and 66^th^ read-out, respectively. In this paper, we optimized k-space sampling among the available settings on our scanner by determining the optimal partial Fourier setting.

### Optimizing Imaging Parameters

Effects of the major imaging parameters (number of readout RF pulses, flip angle, τ, TI, and TD) on GM-WM contrast were simulated using Bloch’s equation based on the values of T_1_, T_2_, and proton density of the WM, GM and CSF of the human brain, which at 3.0 T are 1400/850/3500 ms, 100/90/300 ms [19]–[21], and 0.75/0.65/1.0 [20], respectively. We ignored relaxation effects during RF excitation and assumed perfect spoiling of transverse magnetization after each inversion pulse and before each excitation pulse. Signal intensity and contrast of brain tissues were simulated using Eqs. 2 and 4 in MATLAB (Math Works). Optimal imaging parameters were determined based on simulation results and validated by in vivo experiments.

In our simulations, τ was limited by the hardware (such as acquisition bandwidth and pulse duration time) and total scan time. On our scanner, the largest τ of 10.1 ms corresponds to the narrowest bandwidth of about 140 Hz/pixel for the MP-RAGE sequence. We set τ to 10.1 ms, and simulated the effect of FA on signal intensities and tissue contrasts using Eq. 4 to find the optimal FA. At the optimal FA, signal intensities of GM and WM, and GM-WM contrast were computed as functions of the number of readout RF pulses for different TIs. The functional relationship between GM-WM contrast and TI was simulated to obtain the optimal TI. Finally, GM, WM and CSF signal intensities vs. different TDs were simulated to find the optimized TD.

In the MP-RAGE sequence, the effective inversion recovery time 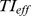 is a major determining factor of image contrast. It is defined as the time interval between the inversion recover pulse and the RF read-out pulse for k-space center. Generally, the readout RF pulse i_max_ corresponding to the maximum image contrast should be used to fill k-space center. The pre-set read-out RF temporal position may not necessarily equal to i_max_. The optimal 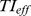 is given by:
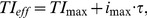
(5)


### Experiments

Ten normal adults (5 males and 5 females) with no known history of neurological diseases participated in the in vivo experiments for MP-RAGE sequence optimization. The mean age of the subjects was 22 years (range: 19 to 25 years). The experimental protocol was approved by the Institutional Review Board (IRB) of the Ohio State University. Additionally, all participants provided written consent before participating in this study.

All human brain images were acquired on a Siemens 3.0 T Trio Tim system with a 32-channel head coil. To test our simulation results, structural brain images were obtained using the MP-RAGE sequence with FOV 232×256 mm^2^, matrix 232×256, slice thickness 1 mm, slice number per slab 160–208, TR/TE = 2200/2.09–4.68 ms, echo spacing time 7.1–10.1 ms, bandwidth 140–240 Hz/pixel, slice partial Fourier 6/8–8/8. Parallel acquisition was conducted in the GRAPPA mode, with reference line phase encoding (PE) = 32, and an acceleration factor of 2. 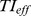 of the MP-RAGE sequence, defined as the time between the inversion recovery pulse and k-space center acquisition, was set at 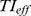 = 880, 900, 920, 950, 960, 1000, 1020 and 1100 ms with a flip angle of 12°. Additionally, brain images were also acquired with 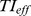 = 950 ms and a number of flip angles ranging from 9 to 14° with 1° increments. The total acquisition time for each brain was about 4 minutes.

In order to compare the quality of images acquired with different imaging parameters, we used the same resolution (FOV 232×256 mm^2^, matrix 232×256, slice thickness 1 mm), phase partial Fourier 6/8, and parallel acquisition parameters (GRAPPA mode, reference line PE = 32, acceleration factor 2) for all the images. Our optimal imaging parameters are: TR/TE = 1950/4.06 ms, 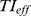
950 ms, flip angle 12°, echo spacing time 10.1 ms, bandwidth 140 Hz/pixel, total scan time 4 minute and 14 second. The default imaging parameters recommended by Siemens are: TR/TE = 2300/2.96 ms, 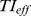 900 ms, echo spacing time 7.1 ms, bandwidth 240 Hz/pixel, total scan time 5 minute and 10 second. The imaging parameters recommended by FreeSurfer [16] are: TR/TE = 2530/3.37 ms, 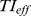 1200 ms, echo spacing time 7.9 ms, bandwidth 200 Hz/pixel, total scan time 5 minute and 41 second. It is noticed that Freesurfer recommended a TI of 1100 ms. We used a TI of 1200 ms by mistake in our experiment. However, additional experiments showed that the WM-GM contrast efficiency of the FreeSurfer protocol is essentially the same when TI = 1100 and 1200 ms. The imaging parameters recommended by ADNI [14] are: TR/TE = 2200/2.96 ms, 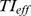 880 ms, echo spacing time 7.1 ms, bandwidth 240 Hz/pixel, total scan time 4 minute and 56 second. All image parameters for different protocols are shown in Table 1. Repeated measurements with identical imaging protocols were conducted on each subject at two different times to estimate noise of the images acquired with each protocol.

**Table 1:** Imaging parameters and CNR efficiencies for our optimized parameters, FreeSurfer, Siemens default, and ADNI.

	TI_eff_(ms)	FA	BW(Hz/pixel)	Slice partialFourier	Echo space(ms)	TR(ms)	TE(ms)	CNR_eff_( 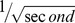 )
FreeSurfer	1200	8	200	1	7.9	2530	3.37	1.39
Siemens	900	9	240	1	7.1	2300	2.96	1.38
ADNI	880	9	240	1	7.1	2200	2.96	1.38
Proposed method	950	12	140	6/8	10.1	1950	4.44	1.90

### Performance Evaluation

In order to quantitatively evaluate image quality, we introduced SNR and CNR efficiencies to evaluate the quality of the images acquired with different imaging parameters because both SNR and CNR are functions of total scan time. The SNR efficiency, 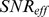, defined as SNR per square root total scan time TA, is:
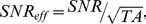
(6)


A single type of tissue may have different signal intensities because of signal inhomogeneity caused by non-uniform transmit field and receive sensitivity. Thus, we cannot use SNR of a single tissue to evaluate image quality. In this paper, global SNR was used as an indicator to evaluate the image quality, avoiding errors caused by signal inhomogeneity.

Similarly, the CNR efficiency is defined as CNR per square root total scan time TA:
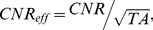
(7)


In order to avoid effects of image inhomogeneity, contrasts between nearby tissues were used to assess image quality. The contrast of GM and WM was estimated using the signal intensity difference between the nearest GM and WM region of interests (ROIs) to avoid effects of signal inhomogeneity. Ten circular ROIs, localized in the frontal lobe, parietal lobe, temporal lobe, and occipital lobe, were chosen to evaluate the image quality with different protocol. For each subject, the ROIs were nearly identical for images acquired with different protocols. They were not exactly the same because images from different protocols did not align perfectly. The radius of each ROI is between 4 to 6 pixels. The distance between each pair of GM and WM ROIs was within 8 pixels. Noise level was determined by subtracting two images acquired with identical imaging parameters at different times. No apparent head motion was observed between images acquired at two different times. The results were also confirmed by a phantom study. We used one-way within-subject design ANOVA to analyze the SNR, and two-way withi- subject design ANOVA to analyze GM/WM CNR with different protocols. Tukey’s posthoc analysis was used to perform pairwise comparisons.

## Results

As shown in Eq. 4, GM-WM contrast is a function of N, TI, τ, θ and the temporal position of the read-out RF pulse. Generally, the total number of RF pulses N is related to the spatial resolution along the slice direction. In vivo experiments confirmed that N was chosen to be 176 for whole brain coverage at a slice thickness of 1 mm on our scanner. To simplify our problem, we set N = 176, FA = 12° and τ = 10.1 ms in our simulation. FA = 12° was chosen based on simulation results shown below. The same procedure can be used for different N’s, FA’s, and τ’s.

**Figure 1:**
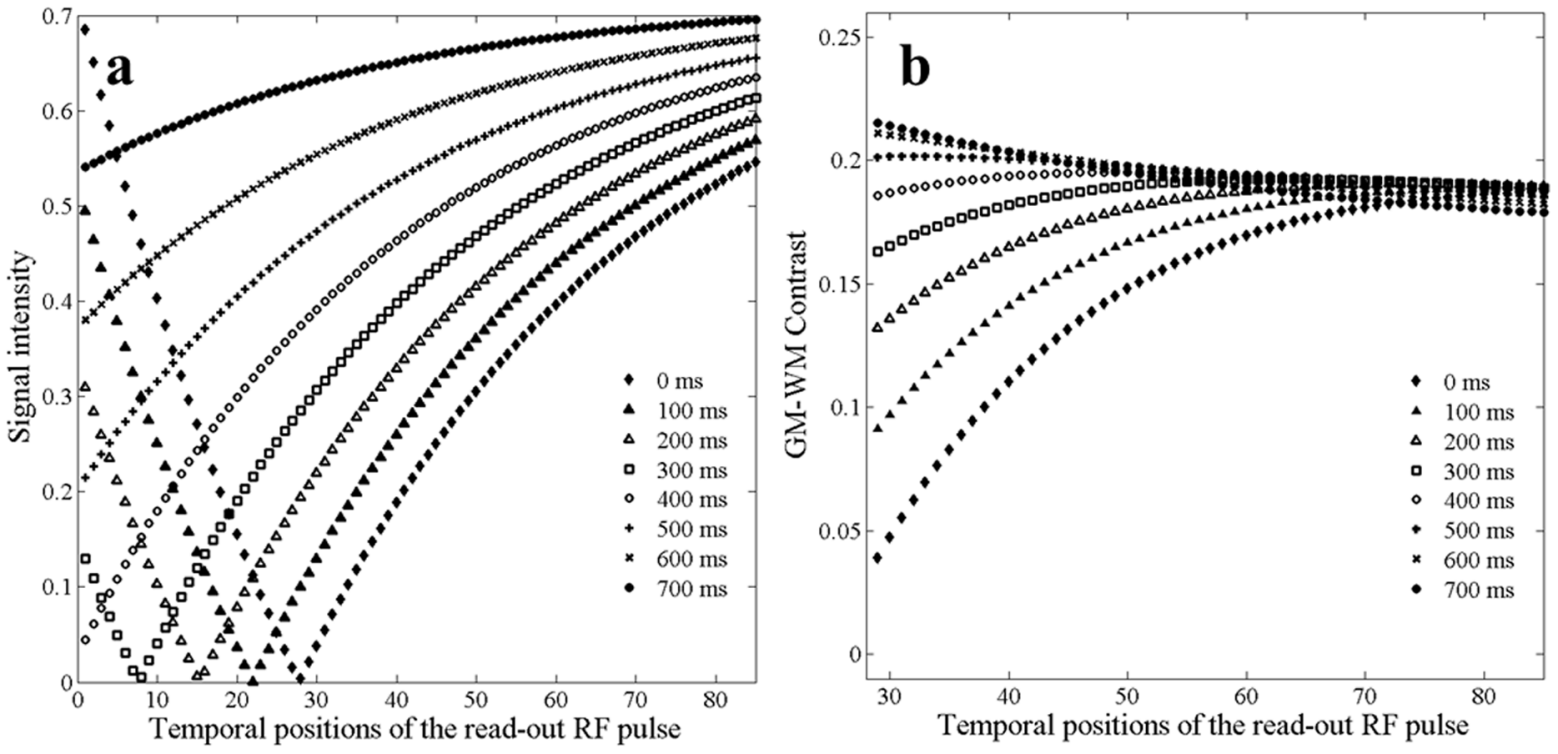
Simulated signal intensity of the CSF and GM-WM contrast for different temporal positions of the read-out RF pulse at different Tis.

Figure 1 shows the simulated signal intensity of the CSF and GM-WM contrast as a function of the temporal position of the read-out RF pulse for different TIs. The SNR of the CSF is illustrated because the signal intensity of the CSF is the lowest among the major brain tissues (CSF, GM and WM) in T_1_-weighted images acquired with the MP-RAGE sequence. If the SNR of the CSF is acceptable, the SNRs of the GM and WM are also acceptable. When the temporal position of the read-out RF pulse is more than 30, the signal intensity of the CSF increases monotonically with increasing TI and the temporal position of the readout RF pulse (Fig. 1**a**). We defined i_max_ as the temporal position of the read-out RF pulse that corresponds to the maximum GM-WM contrast. Fig. 1**b** shows that i_max_ shifts to lower values with increasing TI. i_max_ is not equal to half of the total number of readout RF pulses. That is, the center of the readout RF pulse does not necessarily lead to maximal GM-WM contrast. GM-WM contrast increases but i_max_ decreases with increasing TI. Although the early echoes of the echo train produce greater GM-WM contrast when TI is long, using early echoes to fill low frequency k-space would lead to loss of contrast during the echo train acquisition in the MPRAGE sequence due to relaxation effects [10] and broaden the point-spread function. When we take into account of the tradeoff between GM-WM contrast, CSF signal intensity, and potential loss of contrast, the theoretical i_max_ should be in the range from 30 to 80 for the various TIs. In general, for fixed FA and τ, the GM-WM contrast is a function of N, TI, and the temporal position of the read-out RF pulse; i_max_ is a function of N and TI. For a fixed N, i_max_ is a function of TI.

**Figure 2:**
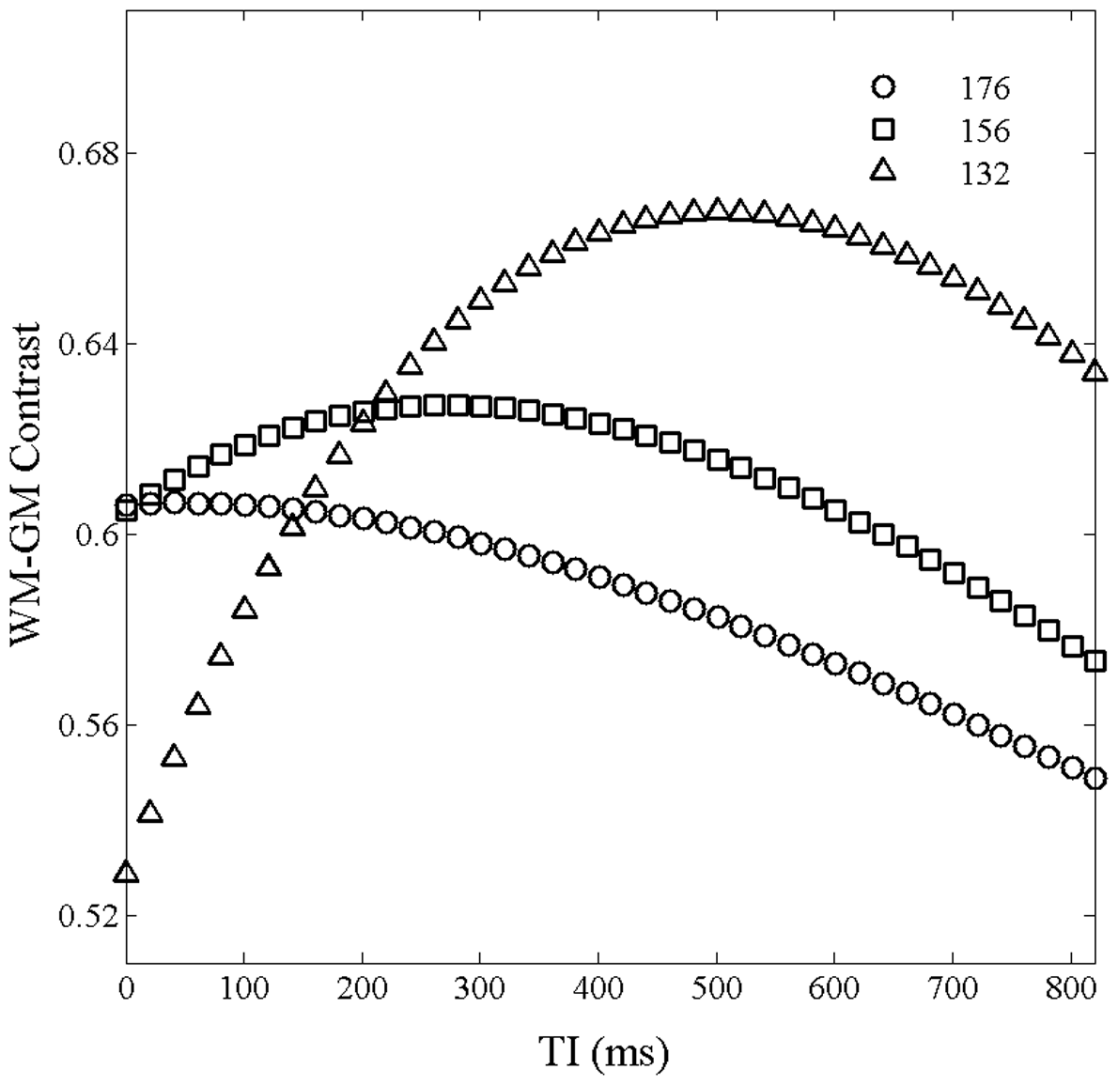
Simulated contrast between the GM and WM as functions of TI for total number of readout RF pulses of 176, 156, and 132. The interval time between readout RF pulses was set to 10.1; the flip angle was set to 12°.

Due to limited options in the product MP-RAGE sequence, we cannot do experiments with the theoretically optimal i_max_. We limited further simulations and experiments to the settings that can be achieved on our scanner. In other words, the choice of real N associated with a nominal N and the temporal position of k-space center are fixed on our scanner. We can only vary TI to obtain maximum GM-WM contrast under the constraints of our scanner. Computer simulations were used to find the optimal TI (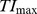) that produces the maximal GM-WM contrast. On our scanner, k-space center is set at the 88^th^, 66^th^ and 44^th^ read-out for a nominal N of 176 with slice partial Fourier of off, 7/8, and 6/8. The simulated GM-WM contrast vs TI curves for different real Ns and corresponding k-space centers are shown in Figure 2. 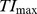 for real N of 176, 156, and 132 were around 50, 300, and 500 ms, respectively. The peaks in the CNR vs TI curves are relatively flat in Fig. 2.

We replaced i_max_ in Eq. 5 with the actual temporal position of the RF-pulse for k-space center and calculated 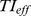. The 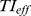 values are 940, 967, and 945 ms for real Ns of 176, 156 and 132, respectively. It is almost a constant for the different real N’s. However, both real N and k-space center strongly affected WM-GM contrast. When the real N is 132 and the k-space center is filled with the 44^th^ RF read-out pulse, the WM-GM contrast was 10% more than that when real N is 176 and k-space center is filled with the 88^th^ RF read-out pulse.

**Figure 3:**
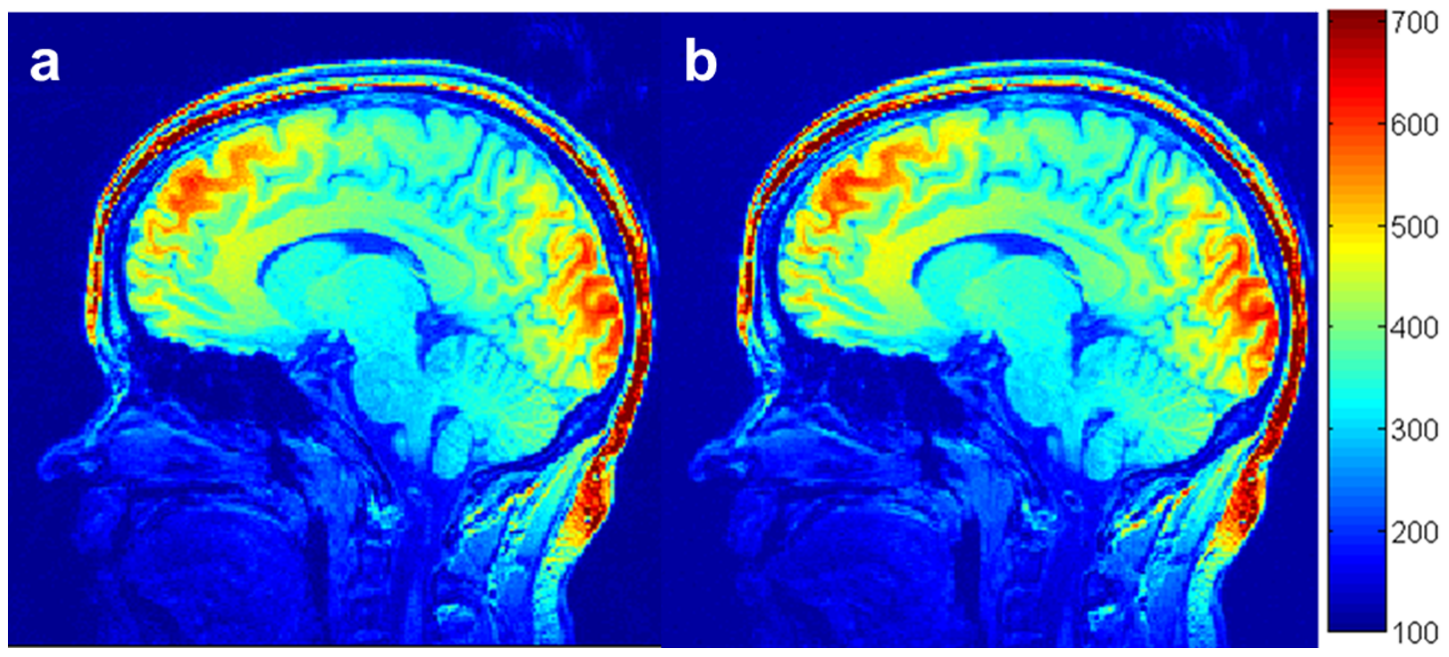
Human brain images acquired with an interval time between readout RF pulses of 10.1°, effective inversion recovery time of 950 ms, total readout RF pulse of 176, and slice partial Fourier factors of 1 (a), and 6/8 (b).

In order to validate our simulation, brain images were acquired without slice partial Fourier (Fig. 3**a**) and with a slice partial Fourier factor of 6/8 (Fig. 3**b**). The other acquisition parameters, including 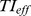, were identical. The results demonstrated that both the SNR and CNR of the images acquired with a slice partial Fourier factor of 6/8 were about 10% higher than those acquired without slice partial Fourier. The results from the in vivo experiments are in good agreement with our simulation results in Fig. 2. Therefore, the optimal real N and k-space center were chosen to be 132 and the 44^th^, respectively, among the realizable settings of our scanner.

**Figure 4:**
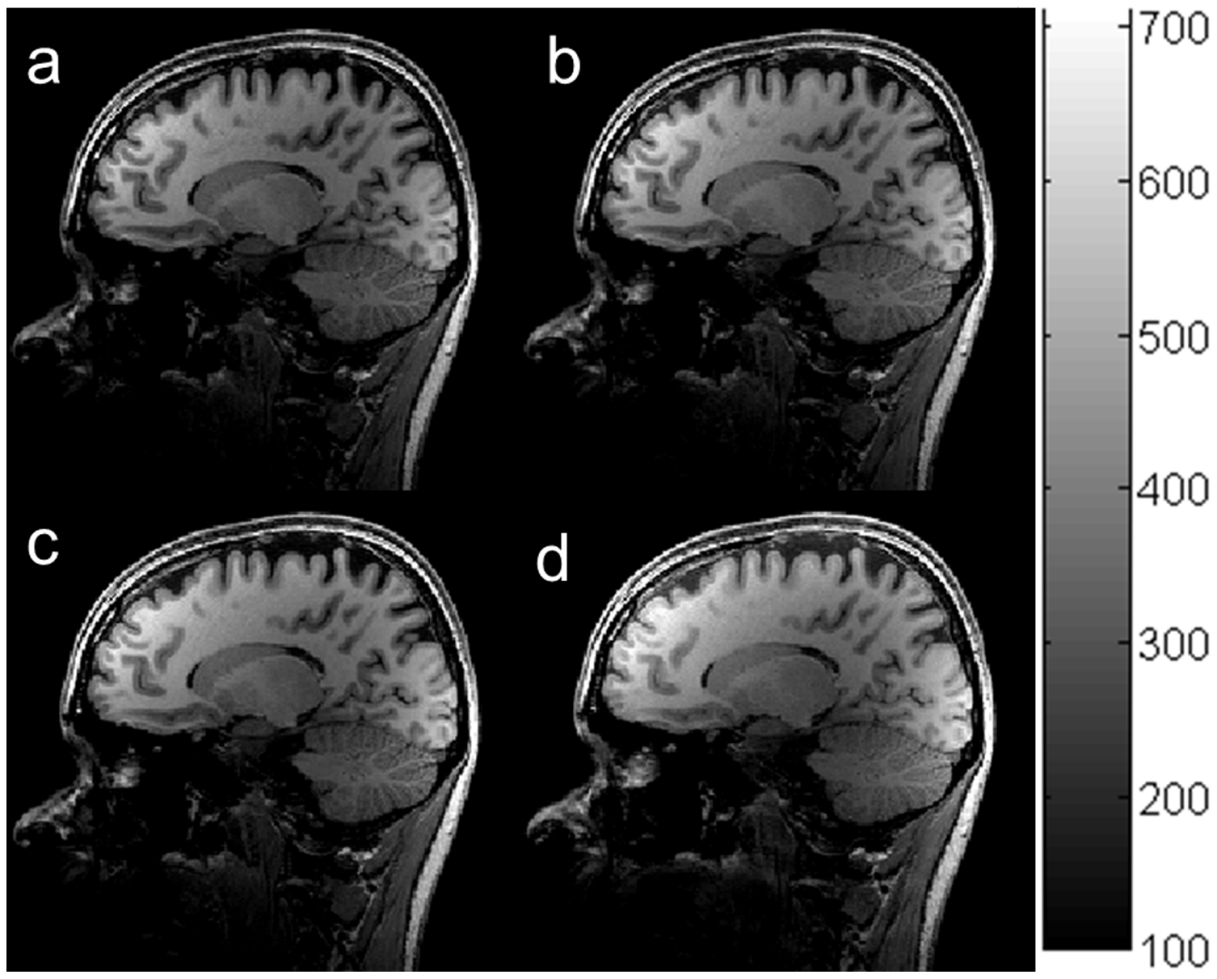
In vivo brain images acquired using the MP-RAGE sequence with different effective inversion recovery times 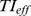: 900 (a), 950 (b), 1020 (c) and 1100 (d) ms at a flip angle of 12°, an interval time between readout RF pulses of 10.1 ms, and slice partial Fourier of 6/8.

Figure 4 shows in vivo brain images acquired using the MP-RAGE sequence with different 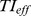
s: 850 (**a**), 950 (**b**), 1000 (**c**) and 1200 (**d**) ms at a flip angle of 12°, with a slice partial Fourier factor of 6/8. The results indicated that the average signal intensity of brain tissues increased from 361 to 421 (around 17%) when 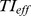 increased from 850 to 1200 ms. The maximum GM-WM contrast occurred at a 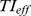 of 980 ms. After k-space optimization, the change in CNR was less than 4% when TI increased from 850 to 1200 ms, in agreement with the simulation results in Fig. 2. To maximize CNR efficiency, the optimal choice of 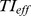 should be short because short 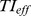 reduces scan time. We set the optimal 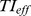 at 950 ms.

**Figure 5:**
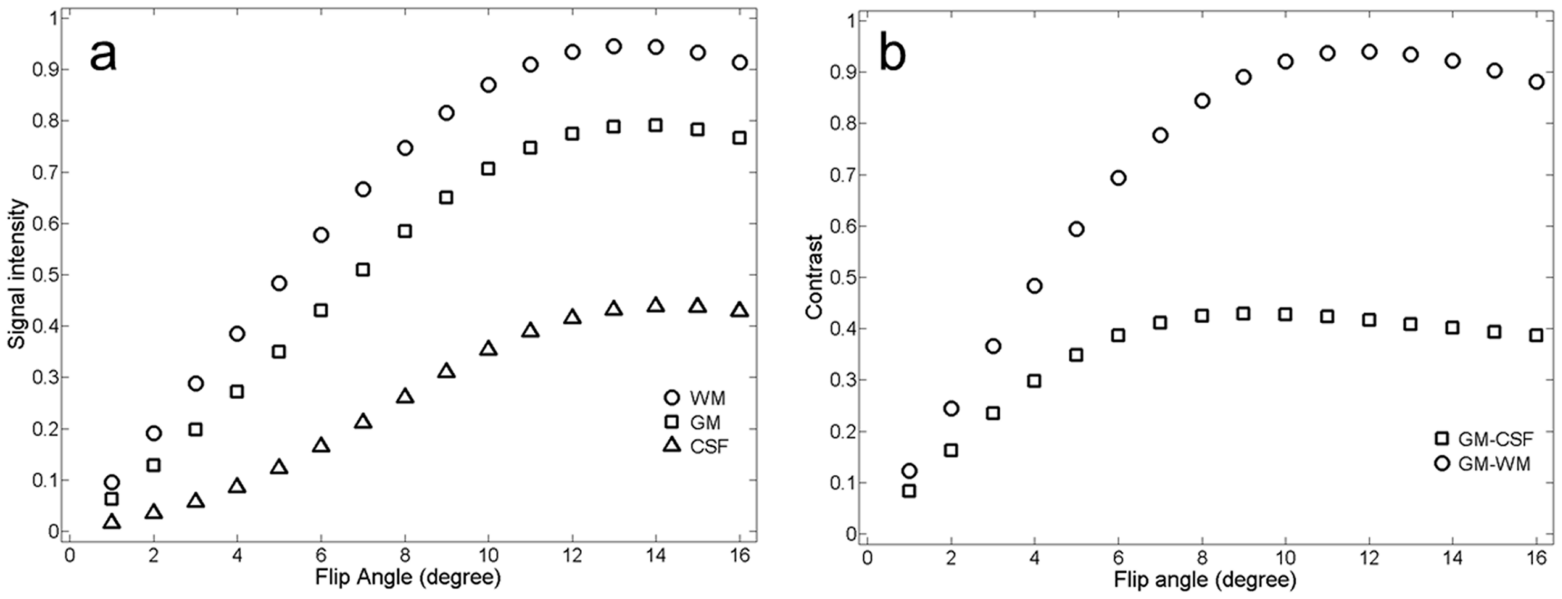
Relationships between simulated signal intensities of brain tissues (the GM, WM and CSF) and flip angle at an interval time between readout RF pulses of 10.1 ms (a) and relationships between simulated contrasts of brain tissues (WM-GM, and GM-CSF) and flip angle under the optimal k-space trajectory (b).

The simulated effects of FA on GM, WM and CSF signal intensities and contrasts between the GM and WM and between the GM and CSF at τ = 10.1 ms are shown in Figure 5. Signal intensity and contrast first increased and then decreased with increasing FA. Signal intensity reached its maximum at FA around 10° for the WM, and around 13° for the GM and CSF. After reaching their maximum values, signal intensities declined slightly with increasing FA. The contrasts started to approach their asymptotic values at FA around 8°, reaching their maxima at FA around 10° and declining slightly at FA around 12°. The contrast curve was almost flat when FA increased from 10° to 12°. That is, variations of FA would have a small impact on the contrast when FA is in the range from 9 to 14°. The signal intensities and contrasts reached their maximum values at different FAs. Since maximizing CNR is more important than maximizing SNR for diagnosis and tissue segmentation, we chose the optimal FA to be 12°. With this FA, GM-WM contrast achieved maximal values and was insensitive to non-uniform FA in different regions of the brain or across brains.

**Figure 6:**
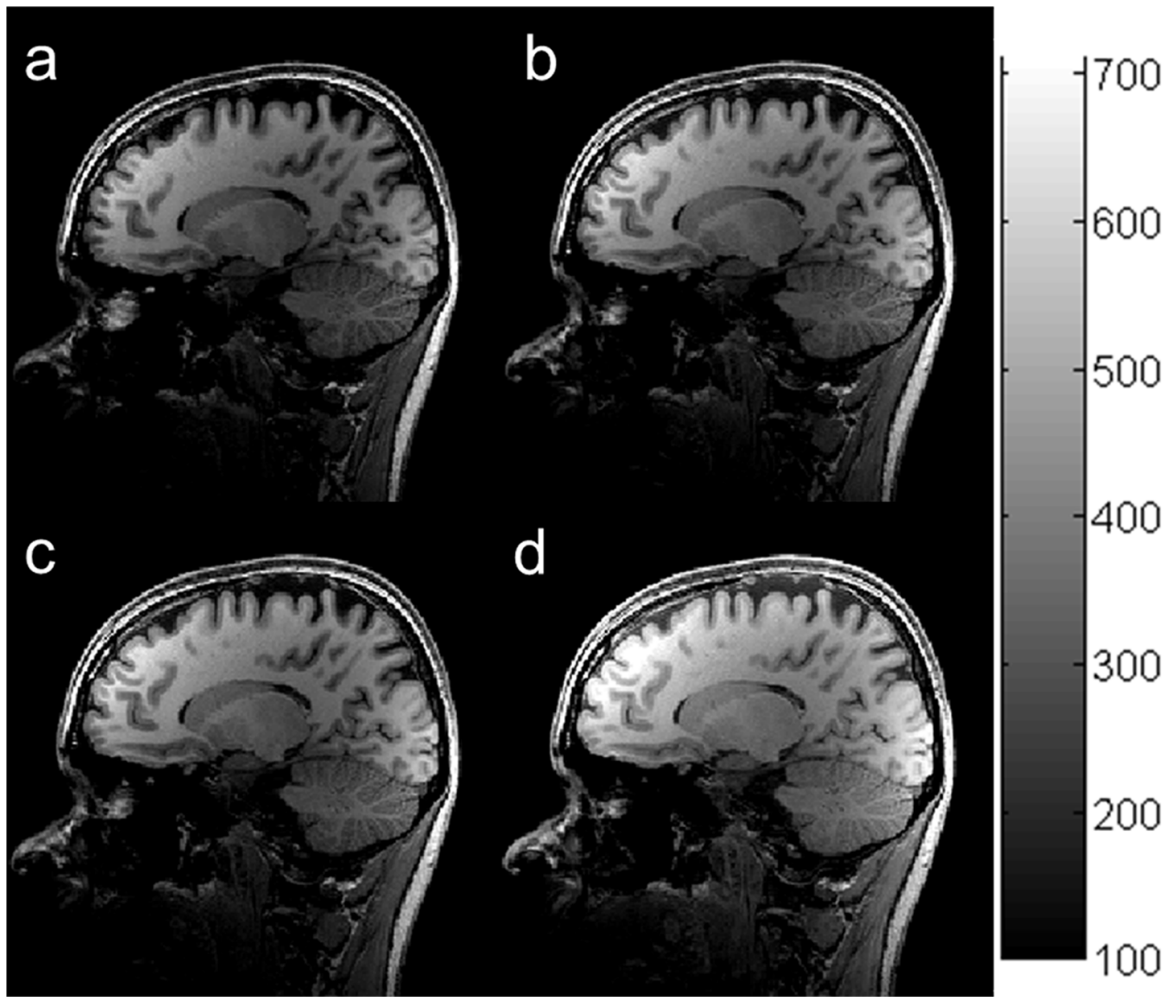
In vivo brain images acquired using the MP-RAGE sequence with different flip angles: 9° (a), 11° (b), 12° (c), and 14° (d) with τ/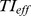/TR = 10.1/950/1950 ms.

Figure 6 shows in vivo brain images acquired using the MP-RAGE sequence at 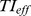 of 950 ms with different flip angles: 9° (**a**), 11° (**b**), 12° (**c**) and 14° (**d**). ROI analysis indicated that with increasing FA, SNRs of the GM and CSF increased, while SNR of the WM increased only when FA was less than 10° and started to decrease when FA was more than 10°. The averaged SNR of brain tissues increased approximately 15% with increasing FA from 9° to 12°. The maximal contrast between the GM and WM occurred at the FA of 12°. These results were completely consistent with the simulation results in Figure 5.

**Figure 7:**
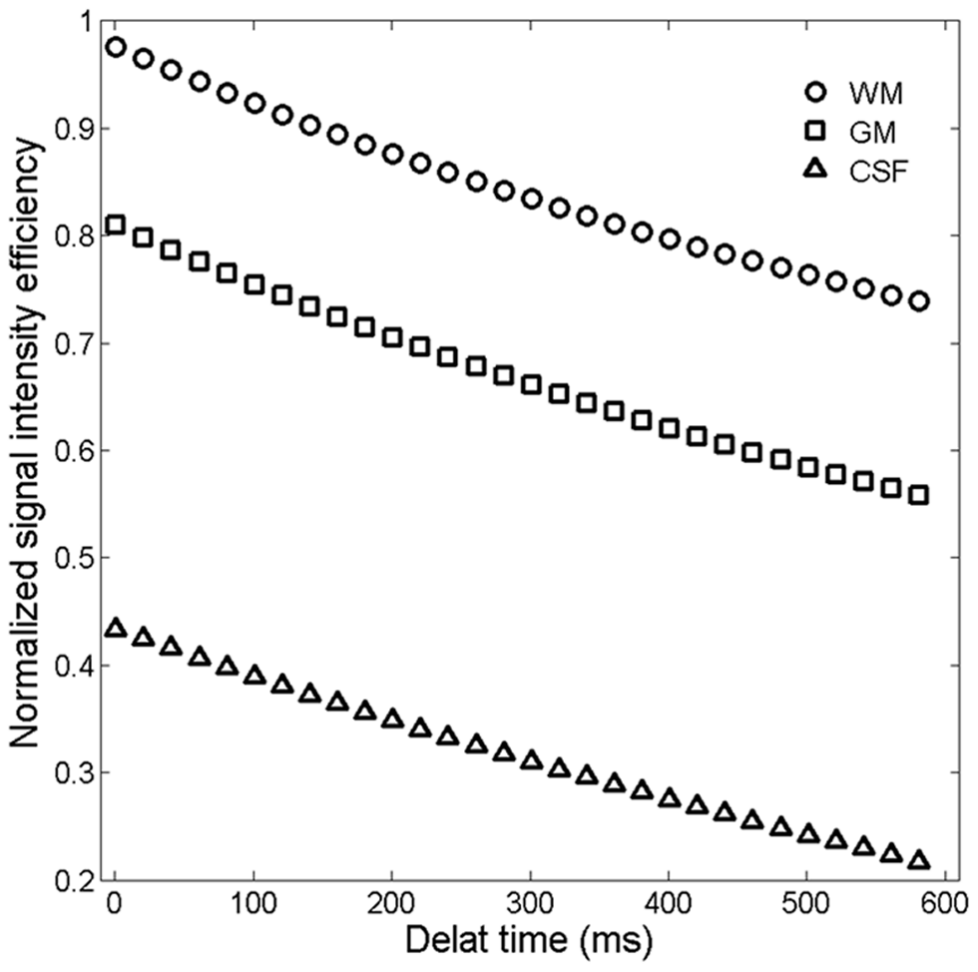
Simulated signal intensities of the GM, WM and CSF at different delay times (TD) at with N = 132 (slice partial Fourier factors of 6/8).

**Figure 8:**
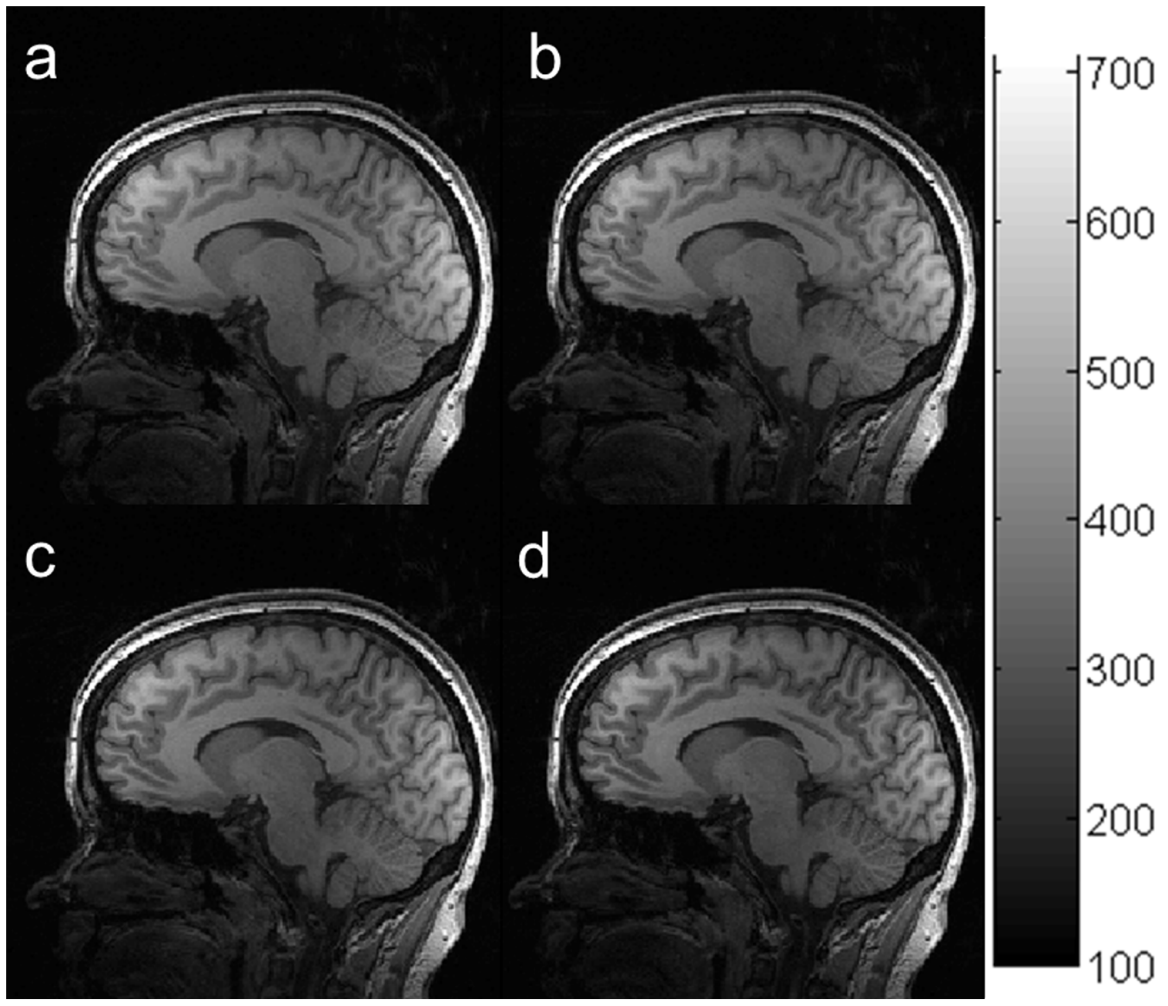
In vivo brain images acquired using the MP-RAGE sequence with different delay times (TD): 0 (a), 50 (b), 100 (c), 200 and 400 (d) ms.

The relationship between signal intensities of brain tissues and TD is shown in Figure 7. All signal intensities of major brain tissues (WM, GM and CSF) decreased with increasing TD. Additionally, the contrasts among these brain tissues increased slightly with increasing TD. In vivo brain images acquired using the MP-RAGE sequence with TD of 0 (**a**), 100 (**b**), 200 (**c**), and 400 ms (**d**) are shown in Figure 8. ROI analysis showed that the SNRs of all brain tissues decreased around 18% when TD increased from 0 to 400 ms. On the other hand, the CNR remained around 38 with increasing TD. The results from the in vivo experiments agreed extremely well with our simulations. This result is inconsistent with other results in the literature that suggested an optimal TD of around 600 ms [12], [13]. The difference may have resulted from different k-space trajectories: non-Cartesian encoding in those studies, and Cartesian encoding in the current study.

**Figure 9:**
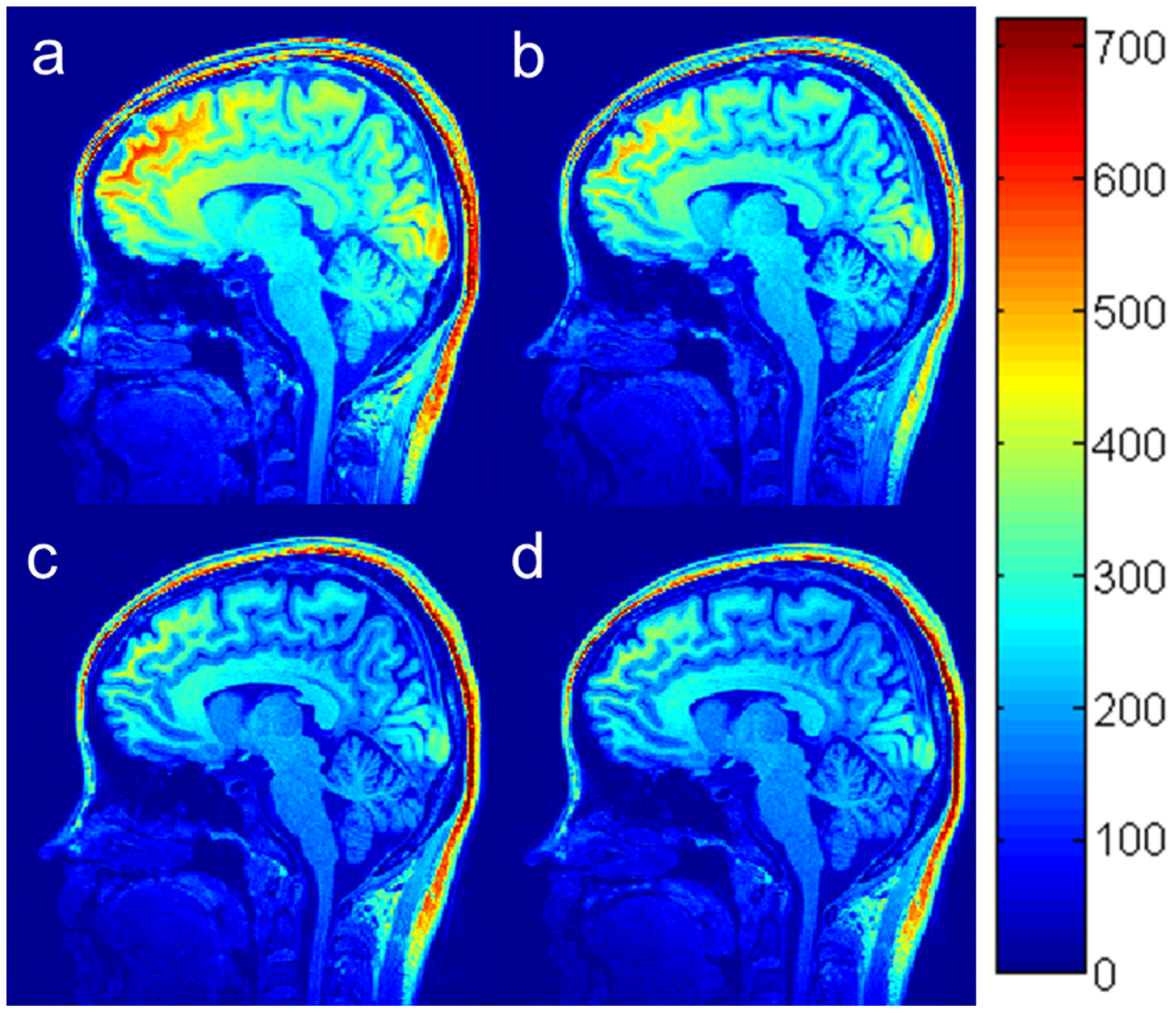
In vivo brain images acquired using the MP-RAGE sequence with different imaging parameters: our optimized parameters (a), FreeSurfer (b), Siemens default (c), and ADNI (d).

In order to evaluate the performance of our optimization procedure, we compared the quality of the images acquired using our optimal setting with those acquired using imaging parameters recommended by Siemens, ADNI [14] and FreeSurfer [16]. The SNR and GM-WM contrast (from the highest to the lowest) over the ten subjects are : 89.1±6.3 and 30.0±1.1 for our optimal imaging parameters (Figure 9**a**), 71.9±5.5 and 25.9±1.1 for recommended parameters in FreeSurfer (Figure 9**b**), 67.7±4.8 and 24.3±2 for Siemens default (Figure 9**c**), and 67.0±4.2 and 24.0±1.7 for ADNI (Figure 9**d**). One-way within-subject design ANOVA on SNR found a significant main effect of protocol (F(3,21) = 66.63, p<0.000001). The mean signal intensity and WM-GM contrast of images acquired with our optimal image parameters was 24% (q = 13.5, p<0.001) and 16% (q = 11.8, p<0.001) higher than those of images acquired using the recommended parameters in FreeSurfer, respectively. The results for our optimal protocol and FreeSurfer protocol are consistent with the simulated results in complementary Figure 1.

**Figure 10:**
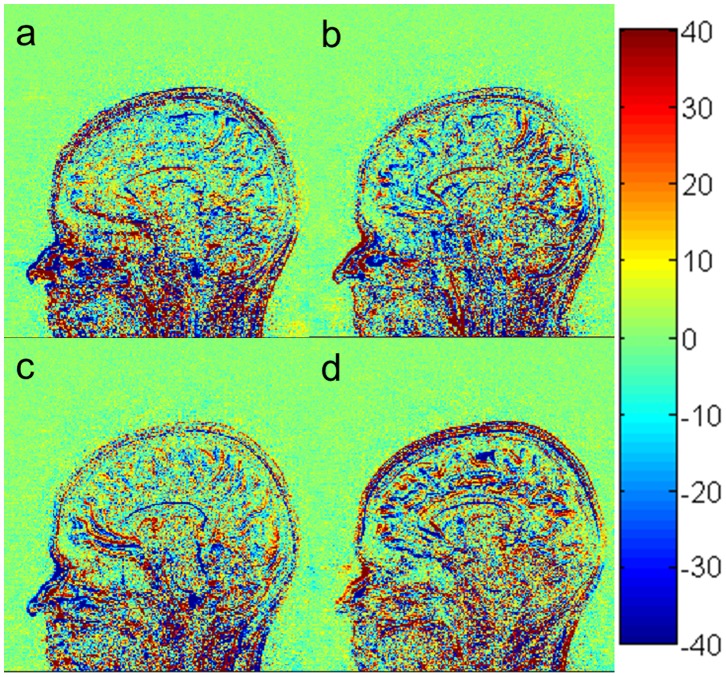
The noise distribution of in-RAGE sequence with different imaging parameters: our optimized parameters (a), FreeSurfer (b), Siemens default (c), and ADNI (d).

To further evaluate the quality of images acquired with different protocols, the noise in images acquired with the different imaging parameters was estimated by subtracting two images acquired with identical imaging parameters at different times [22]. The noise shown in Figure 10 did not have significant difference. Generally, the noise of image acquired at τ of 10.1 ms and receive acquisition bandwidth of around 140 Hz/pixel and a partial Fourier factor of 6/8 (Figure 10**a**) should be significantly lower than that of images acquired with recommended imaging parameters from FreeSurfer, Siemens and ADNI (Figures 10**b**–**d**), because noise caused by the narrow receive acquisition bandwidth is offset by slice partial Fourier. Additionally, we also used the same method to estimate noises of the images of a phantom acquired at different imaging parameters (complementary Figure 2). The two-way within-subject design ANOVA on WM-GM CNR found a highly significant effect of protocol (F(3,21) = 80.32, p<0.000001) but no significant effect of ROI (F(9,63) = 1.475, p>0.15). The WM-GM CNR of the images acquired with our optimal image parameters (Figure 9**a**) was 17% (q = 11.8, p<0.001), 24% (q = 12.7, p<0.001), and 27% (q = 14.8, p<0.001) higher than that of images acquired using the recommended parameters in FreeSurfer (Figure 9**b**), Siemens default (Figure 9**c**), and ADNI (Figure 9**d**), respectively. The total scan time is 4 minute16 second for our optimal protocol, 5 minute 41 second for the FreeSurfer protocol, 5 minute 10 second for the Siemens default protocol, and 4 minute 56 second for the ADNI protocol. The two-way within-subject design ANOVA on WM-GM CNR efficiency found a highly significant effect of protocol (F(3,21) = 173.8, p<5.6 e-15) but no significant effect of ROI (F(9,63) = 1.409, p = 0.2). Thus, the WM-GM CNR efficiency of our optimal protocol is 36% (q = 13.1, p<0.001), 37% (q = 18.7, p<0.001) and 37% (q = 19.3, p<0.001) higher than that of the FreeSurfer, Siemens default, and ADNI protocols.

**Figure 11:**
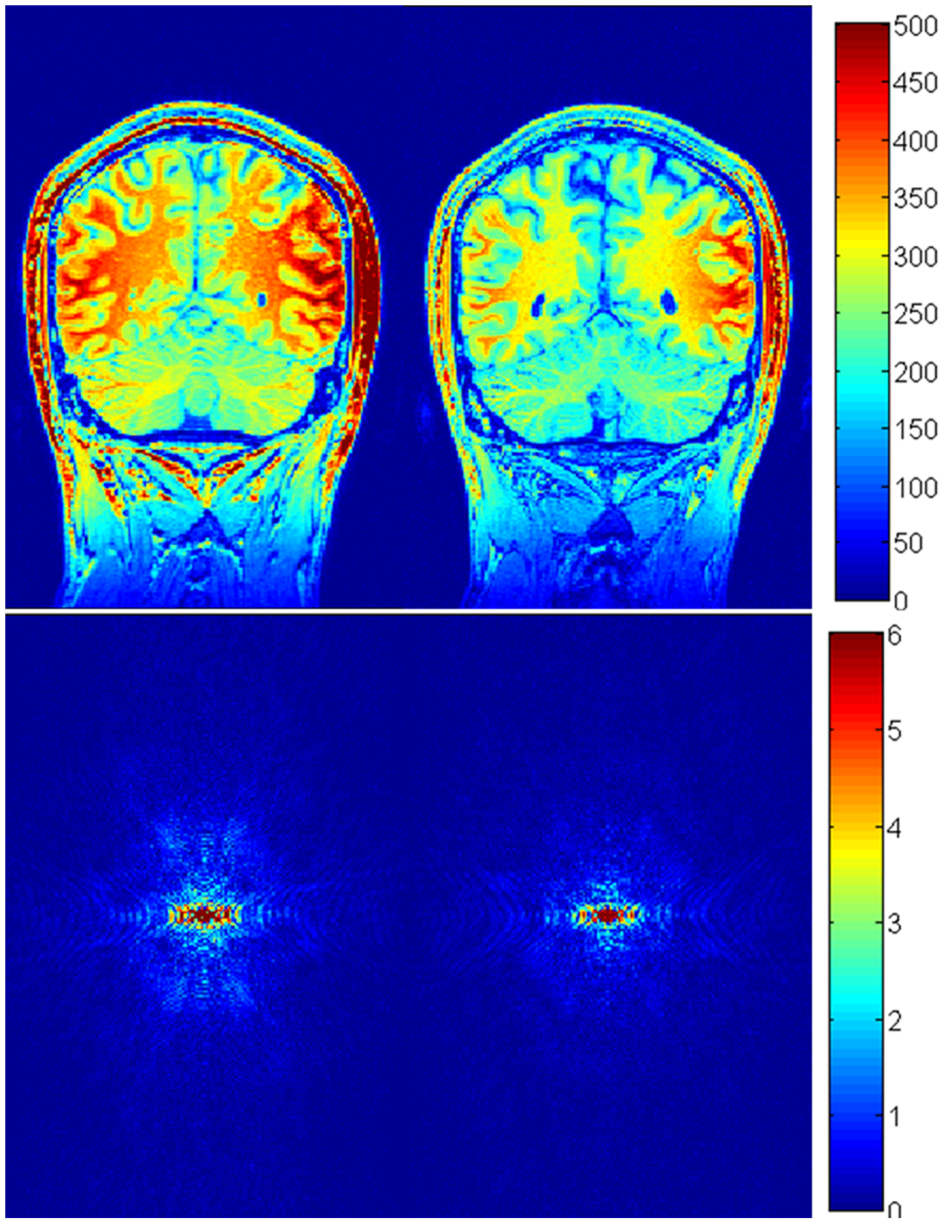
Coronal brain images acquired using the MP-RAGE sequence with our optimized parameters (a) and acquired with imaging parameters recommended by FreeSurfer (b). (**c**) and (**d**) are k-space representations of the images in (**a**) and (**b**), respectively.

**Figure 12:**
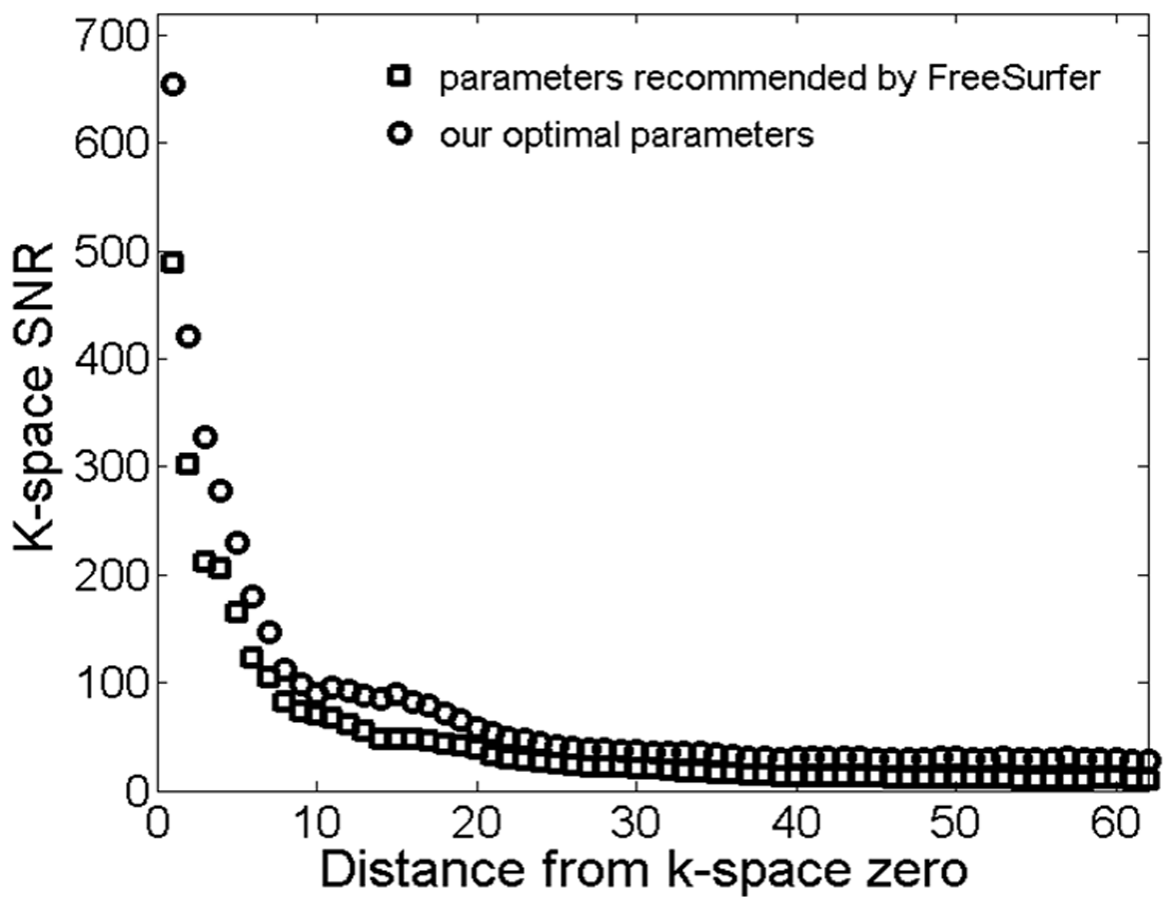
k-space SNR of the images acquired using the MP-RAGE sequence with our optimized parameters and the imaging parameters recommended by FreeSurfer.

In this paper, we optimized k-space sampling and GM-WM CNR efficiency for the MP-RAGE sequence. Although our optimal k-space sampling was aimed at improving low frequency components in k-space, it is necessary to understand the effect of our optimization in the entire k-space. We investigated the frequency domain properties of the images acquired with our optimal imaging parameters and those recommended by FreeSurfer (Figure 11). The magnitude images (Figures 11**b and d**) in k-space were estimated by fast Fourier transformation of the MR images (Figure 11**a and c**). Figure 11**e** is the difference of the images in Figure 11**b and d**. It shows that the magnitude in Figure 11**b** was larger than that in Figure 11**d**, particularly in terms of the low frequency components of the k-space. In other words, our procedure optimized low frequency components without reducing the signal magnitude in the middle and high frequency parts of the k-space. Figure 12 shows, in k-space, the difference of SNR between the images acquired with our optimal imaging parameters and the parameters recommended by FreeSurfer. The noise in k-space was estimated by subtracting two Fourier transformed images acquired with identical imaging parameters at different times. Figure 12 indicated that the SNR with our optimal imaging parameters was higher than that with the parameters recommended by FreeSurfer in k-space.

## Discussion

Application of structural MR brain images in research and clinical settings strongly depends on the quality of the images and image processing algorithms. During structural brain image analyses, large SNR is expect to distinguish tissue from noise, and large CNR is used to distinguish different tissue types. The objective of this paper is to maximize CNR to reduce the intensity overlap of major brain tissues (CSF, GM and WM), and to minimize artifacts through optimizing k-space sampling and imaging parameters.

K-space center is a major determining factor of tissue contrast. The center of the readout RF pulse is generally used to fill the center of the k-space in the MP-RAGE sequence [12], [13]. Our simulations in Figures 1 and 2 and in vivo experiments in Figure 3 suggest that the middle of readout echo train may not correspond to the maximal GM-WM contrast. When N = 176 with and without slice partial Fourier, the optimal k-space center was not in the middle of readout echo train; it occurred earlier – around the 60^th^, 50^th^, 45^th^, 40^th^ read-out RF pulse for slice partial Fourier of off, 7/8,6/8, and 5/8, respectively. The simulated results are shown in complementary Figure 3. Additionally, the simulation and experiments also showed that a smaller total number of readout RF pulses could increase brain tissue contrast. However, the small total number of readout RF pulses may reduce the resolution along the slice direction.

In the MPRAGE sequence, a longer readout echo train can lead to negative consequences such as image blurring and longer TR. Partial Fourier acquisition has usually been used to shorten image acquisition time and echo time (TE) [23], [24]. Here we used partial Fourier acquisition along the slice direction to reduce the total number of readout RF pulses and shift k-space center line to a temporal position that is earlier than the center of the RF pulses in order to increase brain tissue contrast. Although previous studies suggest that changing k-space trajectory and using partial Fourier acquisition may lead to changes to the point spread function (PSF) and introduce additional artifacts [25]–[27], we did not observe any significant increase in noise when we changed k-space trajectory from the center-view to the optimal setting. Our results in Figures 11 and 12 indicate that SNR of the images acquired with our optimal parameters is higher than that of images obtained using parameters recommended by FreeSurfer. This is because (1) although slice partial Fourier acquisition reduces SNR, the narrow acquisition bandwidth in our protocol increased SNR; (2) slice partial Fourier shortens the acquisition time of the last k-space line and total scan time, leading to reduced signal decay and high SNR in high frequency k-space; and (3) the narrow acquisition bandwidth increased readout echo time, optimal flip angle and then SNR. Although a narrow acquisition bandwidth could lead to increased TE and then increased susceptibility artifacts., no difference in susceptibility artifacts was found in images acquired with the different protocols because the T2* of brain tissues at 3.0 T (around 60 ms) is much longer than the typical TE (around 4 ms) of the MPRAGE sequence.

Previous publications on MP-RAGE sequence optimization considered the effects of 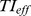, transmit field inhomogeneity, and TD on the SNR and CNR using conventional k-space trajectories and sampling orders [6], [12]–[15]. Optimal k-space center acquisition has received little attention. In most cases, the conventional k-space center acquisition is not optimal because brain tissue contrast is a function of not only 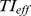 but also total number of readout RF pulses (Figures 1 and 2). The optimal k-space center acquisition may not be at pre-selected positions, such as the beginning or center or end of the readout RF pulses. In this paper, signal intensity and contrast were simulated to find the optimal k-space strategy for the available settings on our scanner. Other imaging parameters, such as FA, 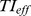, echo space time and TD, were determined by maximizing GM-WM contrast and minimizing artifacts under the optimal k-space strategy. The image quality using our optimal protocol significantly outperformed that using recommended protocols in prior publications (Figures 9 and 10).

We focused on several major imaging parameters in this research. A number of imaging parameters, including echo time, susceptibility artifacts and acquisition bandwidth, that could impact the point-spread function and therefore image quality [25], [28], were not investigated here and should be studied in future research. Several factors can also affect the difference between the initial and final optimal imaging parameters: (1) In the simulation, perfect spoiling was assumed and relaxation during RF pulses and off-resonance artifacts were ignored. With increasing flip angle, perfect spoiling becomes difficult [29]. As a result, imperfect spoiling affects the accurate estimation of signal intensity and other parameters, such as relaxation time. Further studies of in vivo experiments at different flip angles indicated that artifacts and noise were most or less constant for flip angles from 8 to 14°. If the study objective is to segment brain tissues and quantify their volumes, accurate estimation of signal intensity is not very important. The effect of imperfect spoiling can be ignored. However, imperfect spoiling is a big problem if the images acquired at large flip angles are used to estimate relaxation time [21]. In that case, Eq. 2 cannot be used to estimate relaxation time precisely. (2) The MR parameters of brain tissues, such as relaxation times and proton densities, vary across different brain regions of a single subject and across brains of different subjects. The variability was ignored in the simulation; average MR parameters were used to estimate the initial optimized imaging parameters in the simulation. Thus, in vivo experiments should be used to refine the optimal imaging parameters following simulation. In other words, the simulation provided the range of the optimal imaging parameters and shortened the time for the optimization of MP-RAGE sequence. Such simulation provided an excellent tool for MR sequence optimization, reducing the cost of implementing untested prototypes on actual MRI systems.

In comparison to previous MP-RAGE protocols, we (1) improved SNR and CNR by more than 15% when we increased echo-spacing from 8 to 10.1 ms and FA from 9 to 12° (Fig. 7) (2) optimized k-space trajectory and improved CNR by more than 10%, (3) minimized TD and enhanced the efficiency of MP-RAGE sequence, and (4) reduced the total number of readout RF pulses using slice partial Fourier and slightly increased SNR and CNR. As a result, an increase in CNR efficiency of around 35% was achieved through the combination of the optimizations of the various aspects of the MP-RAGE protocol.

## Conclusions

Optimization of the imaging parameters of the MP-RAGE sequence for human brain imaging was performed using computer simulations. We conclude: (1) The relationship between tissue contrast and 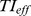 depends strongly on k-space sampling. Optimal k-space center acquisition should not be limited to certain fixed RF readout pulses but be determined by maximum tissue contrast. K-space sampling was optimized for available settings on our scanner. (2) SNR decreases but CNR increases slightly with increasing TD. The minimized TD can increase the efficiency of image acquisition. (3) The optimal imaging parameters are TD of 0 ms, 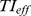 of around 950 to 1000 ms, τ of 10.1 ms, and flip angle of 12°. (4) Slice partial Fourier reduces the total number of readout RF pulses and shifts low-frequency k-space acquisition and therefore improves image contrast, and (5) compared with previous results, our optimized parameters increased CNR efficiency by about 35%. The simulation results agreed excellently with the data from in vivo experiments. Using computer simulation for sequence optimization is time and cost efficient compared to in vivo experiments. The proposed optimization methodology can be easily extended for different human organs, field strengths, image reconstruction, and MR sequences.
